# DynG: a dynamic scaling factor for thermographic stomatal conductance estimation under changing environmental conditions

**DOI:** 10.1111/nph.70555

**Published:** 2025-09-06

**Authors:** Jiayu Zhang, Elias Kaiser, Leo F. M. Marcelis, Silvere Vialet‐Chabrand

**Affiliations:** ^1^ Horticulture and Product Physiology, Department of Plant Sciences Wageningen University & Research Wageningen 6708 PB the Netherlands; ^2^ Research Institute of Agriculture and Life Sciences Seoul National University Seoul 08826 Republic of Korea

**Keywords:** *Arabidopsis thaliana*, conductance index, dynamic environment, energy balance, high‐throughput phenotyping, monitoring, stomatal conductance, thermal imaging

## Abstract

- Thermal imaging is a key plant phenotyping and monitoring technique but faces major bottlenecks in accurately and efficiently inferring stomatal conductance (*g*_sw_) from leaf temperature. The conductance index (*I*_g_) was previously proposed to estimate *g*_sw_ from thermography by linking temperature differences between real and artificial leaves (ALs) based on the leaf energy balance. However, *I*_g_ is highly sensitive to environmental fluctuations, hampering interpretation and reducing reproducibility.
- We developed a simple and novel correction factor (named DynG) for *I*_g_ that accounts for environmental fluctuations when scaling *I*_g_ to *g*_sw_. This was achieved by capturing temperature variations in a set of ALs with a range of known constant pore conductances. This approach provided the *I*_g_–conductance relationship, using ALs as a reference, to infer *g*_sw_ of real leaves from their measured *I*_g_.
- In fluctuating environments, *g*_sw_ estimated using DynG showed greater accuracy and stability than *g*_sw_ calculated from *I*_g_ alone, and was in good agreement with *g*_sw_ determined using lysimetric and gas exchange methods. DynG's power was further showcased in distinguishing *g*_sw_ of Arabidopsis genotypes differing in stomatal traits (Col‐0, *epf1epf2*, and EPF2OE).
- We conclude that *I*_g_ corrected with DynG can reliably estimate *g*_sw_ in fluctuating environments without complex modeling, opening new avenues for *g*_sw_ phenotyping and monitoring.

## Introduction

Plants constantly exchange energy and matter with their environment (Lösch, 1994; Jones, 2013). A critical process is the regulation of leaf gas exchange, including water vapor. Stomata are size‐adjustable pores mostly found on leaf surfaces that regulate the balance between carbon assimilation by photosynthesis and water loss through transpiration (*E*, mol m^−2^ s^−1^). Dynamic regulation of the stomatal aperture is achieved via changes in guard cell turgor pressure, thereby changing stomatal conductance to water vapor (*g*_sw_) (Jones, 1998; Yu *et al*., 2004). *g*_sw_ is a complex trait influenced by environmental signals (e.g. light intensity and spectrum, air humidity, temperature, and CO_2_ concentration; Casson & Hetherington, 2010; Casson & Gray, 2008), genetics (Pantin *et al*., 2013; Yu *et al*., 2015), circadian rhythms (Webb, 2003; Dodd *et al*., 2004), and acclimation to the growth environment (McElwain *et al*., 2016; Matthews *et al*., 2018; Habermann *et al*., 2019). The considerable inter‐ and intraspecies variation of *g*_sw_ (Ohsumi *et al*., 2007; McAusland *et al*., 2016; Xiong *et al*., 2018; Faralli *et al*., 2019) can be measured by phenotyping large populations (Prado *et al*., 2018) which, combined with genetic mapping technology, can help identify candidate genes. Recently, Pignon *et al*. (2021) selected genes related to high water use efficiency by monitoring stomatal movement with thermography in a cabinet with stable environmental conditions. However, when scaling up to high‐throughput phenotyping (HTP) platforms in which local microclimate heterogeneity is inevitable, quantifying *g*_sw_ across large‐scale genotypes under fluctuating conditions becomes a critical challenge. Additionally, *g*_sw_ is a main determinant of crop yield (McAusland *et al*., 2023), and continuous monitoring of *g*_sw_ is critical for fine‐tuning growth conditions and maximizing productivity in controlled‐environment agriculture (Salvatori *et al*., 2021). However, its practical application remains constrained by the lack of suitable and easy‐to‐use methods, sensors, and models (Kaiser *et al*., 2024).

The most commonly used method for assessing *g*_sw_ involves measurements using porometers or infrared gas analyzers that require enclosing the leaf in a chamber. While such measurements are generally accurate, they are lengthy, isolate the leaf from its usual environment, and require relatively expensive equipment (Mayanja *et al*., 2024). Gravimetric methods (Cirelli *et al*., 2012) also allow for accurate *g*_sw_ estimation under controlled environments, as long as boundary layer conductance is known. Other techniques, including microscopy (Sun *et al*., 2021) and Chl fluorescence (Takaoka *et al*., 2020), can provide useful measures but are limited in terms of accuracy, throughput, and/or environmental sensitivity. Noncontact thermal imaging is ideal for automatically phenotyping and monitoring *g*_sw_ of many plants, as variations in leaf or canopy temperature are affected by E, which can be used to estimate *g*_sw_ (Costa *et al*., 2013; Driever *et al*., 2023). Early studies calculated *g*_sw_ using a full leaf energy balance model that accounted for energy fluxes and mass transfer between the leaf and its microclimate (Jones, 2004). To simplify the application of the leaf energy balance model, Jones (1999b) designed wet and dry reference materials (called ‘references’ hereafter) with similar radiative and aerodynamic properties as real leaves. References can eliminate the need for measuring several environmental parameters (e.g. short‐ and long‐wave radiation) (Leinonen *et al*., 2006; Guilioni *et al*., 2008); however, fluctuations in the surrounding environment may compromise their accuracy (Maes & Steppe, 2012). Vialet‐Chabrand & Lawson (2019, 2020) further proposed a thermography‐based method that allows for the continuous monitoring of *g*_sw_ variations, using any reference material under fluctuating conditions. However, the reliance of the method introduced by Vialet‐Chabrand & Lawson (2019, 2020) on a dynamic leaf energy balance model introduces complexity, limiting its broader adoption. Therefore, a method that is simple, accurate, and robust for estimating *g*_sw_ under dynamic environments is highly desirable.

The conductance index (*I*_g_) is based on temperature differences between actual and reference leaves (Jones, 1999a):

(Eqn 1) $$I_{g}=\frac{T_{\mathrm{dry}}-T_{\text{leaf}}}{T_{\text{leaf}}-T_{\mathrm{wet}}}$$

where *T*_dry_ is the temperature of a dry (nontranspiring) reference, and *T*_wet_ is the temperature of a wet (fully transpiring) reference, representing the temperatures corresponding to a maximum range of evaporation in a given environment. *I*_g_ is linearly related to *g*_sw_ and scaled using the variable *G*, which is a function of environmental conditions and leaf anatomy (Maes *et al*., 2016; Pignon *et al*., 2021; Savvides *et al*., 2022):

(Eqn 2) $$I_{g}=g_{\mathrm{sw}}\times G$$

Under a steady environment, *G* can be considered constant, allowing *g*_sw_ to be calculated from *I*_g_. However, when environmental conditions (e.g. wind speed, air relative humidity, or temperature) change, so do *G* and *I*_g_ (Maes & Steppe, 2012; Vialet‐Chabrand & Lawson, 2020), making it impossible to accurately compute *g*_sw_. Even in controlled environments, spatial heterogeneity in the microclimate around plants occurs due to uneven air mixing, increasing the difficulty of *I*_g_ application (Boulard *et al*., 2004; Poorter *et al*., 2012). An additional problem is that the *I*_g_–*g*_sw_ relationship is a simplification based on the assumption that both actual and reference leaves evaporate either from both sides simultaneously or from one side only (Guilioni *et al*., 2008). In other cases (i.e. leaf and references transpiring from different sides), *g*_sw_ cannot be directly derived from *I*_g_, and the uncertain contribution of boundary layer conductance to water vapor (*g*_bw_) plays a role in correcting for anatomical differences between actual and reference leaves (Leinonen *et al*., 2006). While *I*_g_ offers operational simplicity as an indicator of *g*_sw_, its sensitivity to environmental fluctuations and the influence of morphological differences on *g*_bw_ limit the applicability of *I*_g_.

Vialet‐Chabrand & Lawson (2020) proposed using a set of at least three artificial leaves (ALs) with known pore conductance and similar leaf properties, along with ‘wet’ and ‘dry’ references, for deriving *G* dynamically and converting *I*_g_ into *g*_sw_ under any environmental condition. As this idea was never experimentally validated, we explored it further by developing a dynamic scaling factor called DynG and assessing its application under several environmental conditions. The pore conductance of AL was first characterized using microscopy and gas exchange measurements. Then, three *Arabidopsis thaliana* genotypes differing in stomatal density and size, resulting in large differences in *g*_sw_ (Franks *et al*., 2015), were used to test the performance of DynG. Results were validated against a lysimetric method that tracked transpiration‐driven changes in plant weight. Our method solves a critical bottleneck in plant‐ or canopy‐scale *g*_sw_ phenotyping and monitoring.

## Materials and Methods

### Theory

Stomatal conductance to water vapor (*g*_sw_) can be expressed using leaf temperature (*T*_leaf_) and environmental factors based on the leaf energy balance model (Leinonen *et al*., 2006):

(Eqn 3) $$g_{\mathrm{sw}}=\frac{\rho_{\mathrm{air}}\times C_{p}\times \left[\left(T_{\text{leaf}}-T_{\mathrm{air}}\right)\times s+\mathrm{VPD}\right]}{\gamma \times \left[R_{\mathrm{ni}}+\left(T_{\text{leaf}}-T_{\mathrm{air}}\right)\times \rho_{\mathrm{air}}\times C_{p}\times g_{\mathrm{HR}}\right]}-\frac{1}{g_{\mathrm{bw}}}$$

where *ρ*_air_ is air density (kg m^−3^), *C*_p_ is the specific heat capacity of air (J kg^−1^ K^−1^), *T*_air_ is air temperature, *s* is the slope of the curve relating saturation water vapor pressure to *T*_air_, VPD is air vapor pressure deficit (Pa), *γ* is the psychrometric constant (Pa K ^−1^), *R*_*ni*_ is net isothermal radiation (W m^−2^), *g*_HR_ is boundary layer conductance to heat and radiative transfer (*g*_HR_ = 0.92 × *g*_bw_ + *g*_R_), with *g*_R_ being a radiative conductance (*g*_R_ = $\frac{4\times \varepsilon_{l}\times \sigma \times T_{\mathrm{air}}^{4}}{C_{p}}$), *σ* is the Stefan–Boltzmann constant (W m^−2^ K^−4^), and *ε*_l_ is leaf long‐wave radiation emissivity (Jones, 2004). Assuming that dry and wet leaves have similar optical and aerodynamic properties to real leaves, they can be used to eliminate *R*_*ni*_ and *D*, and Eqn 3 can be simplified to:

(Eqn 4) $$g_{\mathrm{sw}}=\frac{T_{\mathrm{dry}}-T_{\text{leaf}}}{T_{\text{leaf}}-T_{\mathrm{wet}}}\times \frac{g_{\mathrm{bw}}\times g_{\mathrm{HR}}}{g_{\mathrm{HR}}+g_{\mathrm{bw}}\times \frac{s}{\gamma }}=I_{g}\times \frac{1}{G}$$

**Fig. 1:**
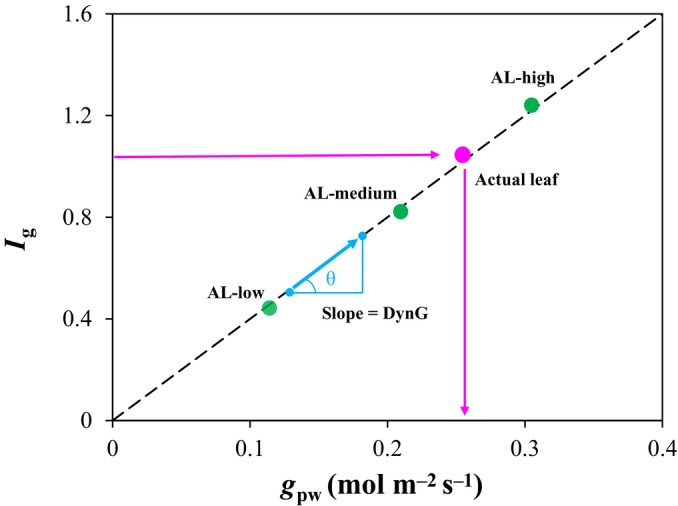
Schematic diagram of DynG. AL‐Low, AL‐Medium, and AL‐High are artificial leaves with different known pore conductance (*g*_pw_, mol m^−2^ s^−1^). Note that data points are fictitious. The slope *θ* of the relationship between *I*_g_ and *g*_pw_ represents DynG (blue arrows). Pink arrows indicate the projection of the actual leaf data point onto the *x*‐ and *y*‐axes.

The value of *G* that determines the *g*_sw_–*I*_g_ relationship is complex and mainly affected by two conductances: *g*_bw_ and *g*_HR_, suggesting *G* is sensitive to changes in the environment (wind speed and *T*_air_). In addition, *G* is sensitive to variation in VPD due to the *s* and *γ* terms. To address this problem, we used three AL with different pore conductances (*g*_pw_) to establish a linear relationship between *I*_g_ and *g*_pw_ (Fig. 1) and derive *G* dynamically. Note that in AL, the conductance to water vapor diffusion is considered constant, unlike in real leaves in which stomatal movement causes *g*_sw_ to change; therefore, we termed it *g*_pw_ instead of *g*_sw_ whenever referring to conductance in AL. The slope between the *I*_g_ and *g*_pw_ relationship was named ‘DynG’, which can replace ‘*G*’ in further equations. *I*_g_ of actual leaves can theoretically be derived from this relationship under a large range of environmental conditions.

The linear relationship (Eqn 4) between *g*_sw_ and *I*_g_ can only be maintained when the wet AL matches the stomatal ratio (SR; ratio of stomatal densities between the ad‐ and abaxial leaf sides) of an actual leaf (when SR = 0 or 1). This ensures accurate simulation of transpiration patterns and boundary layer characteristics between artificial and actual leaves. In the case of anisolateral leaves (SR = 0–1), there is a difference between *g*_sw_ and *g*_bw_ on the upper and lower surfaces, and thus, no explicit expression for *g*_sw_ can be inferred from *I*_g_. By contrast, *I*_g_ is a linear function of total leaf conductance to water vapor (*g*_tw_), which is an integrated expression that combines adaxial and abaxial *g*_sw_ with *g*_bw_ (Guilioni *et al*., 2008). When using an AL wetted on one side, the relationship between *g*_tw_ and *I*_g_ is:

(Eqn 5) $$g_{\mathrm{tw}}=\frac{1}{\frac{\text{DynG}}{I_{g}}+\frac{1}{g_{\mathrm{bw}}}}$$

SR determines the relative contributions of the upper and lower epidermis in transpiration and *g*_sw_ (Jalakas *et al*., 2024) and can be used to derive *g*_sw_ from *g*_tw_, as described in the manual of the Li‐6800 photosynthesis system (Li‐Cor Biosciences, Lincoln, NE, USA; https://www.licor.com/env/support/LI‐6800/manuals.html):

(Eqn 6) $$g_{\mathrm{sw}}=\frac{2}{\left(\frac{1}{g_{\mathrm{tw}}}-\frac{1}{g_{\mathrm{bw}}}\right)+\sqrt{{\left(\frac{1}{g_{\mathrm{tw}}}-\frac{1}{g_{\mathrm{bw}}}\right)}^{2}+\frac{4\mathrm{SR}}{{\left(\mathrm{SR}+1\right)}^{2}}\times \left(\frac{2}{g_{\mathrm{tw}}}-\frac{1}{g_{\mathrm{bw}}}\right)\times \frac{1}{g_{\mathrm{bw}}}}}$$

When SR = 1, Eqns 5, 6 can be simplified (Guilioni *et al*., 2008):

(Eqn 7) $$g_{\mathrm{sw}}=\frac{2\times I_{g}\times g_{\mathrm{bw}}}{I_{g}+2\times \text{DynG}\times g_{\mathrm{bw}}}$$

*g*_sw_ can be calculated if DynG and *g*_bw_ are known. *g*_bw_ can be estimated using the leaf energy balance with dry and wet AL (see Eqn 10, for details). In this study, the value of *g*_sw_ includes both stomatal and cuticular components, as the thermal imaging approach does not allow for their separation. However, the contribution of cuticular conductance is typically negligible compared to that of stomatal conductance (Márquez *et al*., 2022). Gas exchange methods (LI‐6800) also estimate *g*_sw_ as the sum of stomatal and cuticular conductance.

### Experimental materials

#### Plant material

Seeds of *A. thaliana* (L.) Heynh Col‐0 (wild‐type (WT)), epidermal patterning factor (EPF) double mutants (*epf1epf2*) with increased stomatal density relative to WT as well as the EPF2‐overexpressor (EPF2OE) with reduced stomatal density relative to WT (Hara *et al*., 2009; Hunt & Gray, 2009) were placed in Petri dishes lined with moist filter paper at 4°C in a dark room for stratification. After 3 to 4 d, seeds were transferred to pots (175 cm^3^, 5 × 5 × 7 cm) filled with a standard sterilized substrate (Lensli, Bleiswijk, the Netherlands), and pots were sealed with black polyethylene plastic films to prevent soil evaporation. Pots were moved to a controlled‐environment climate chamber at a photoperiod of 10 h, *T*_air_ of 23°C : 20°C (day : night), RH_air_ of 70% ± 2%, and ambient CO_2_ of *c*. 450 ppm. Plants were grown at an average light intensity of 150 μmol m^−2^ s^−1^ provided by GreenPower LED deep red/blue modules (Signify, Eindhoven, the Netherlands). At 21 to 28 d after germination, plants with uniform growth were used for experiments. Three biological replicates per genotype (*n* = 3) were used for all measurements.

#### Construction of AL

**Fig. 2:**
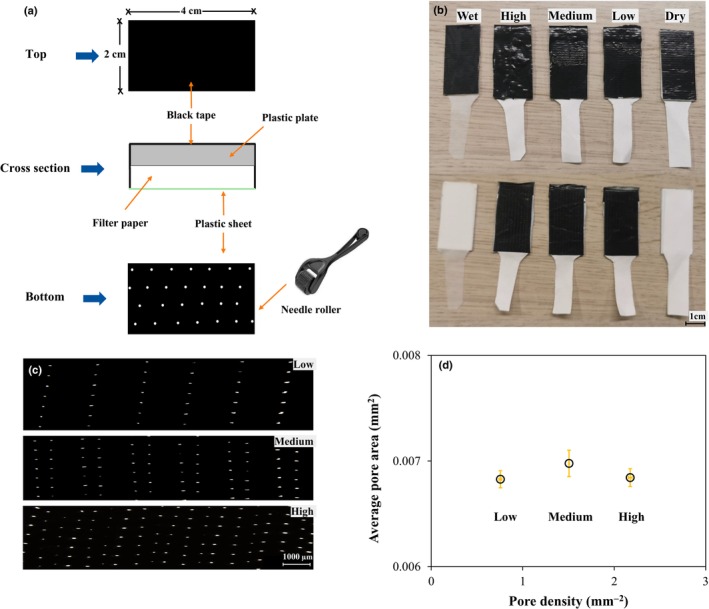
Artificial leaves (ALs) with known pore conductance. (a) Schematic of AL including black tape, plastic plate, filter paper, and porous plastic film (thickness = 0.02 mm). (b) Top and bottom sides of five AL. From left to right: wet, high (*g*_pw_high_), medium (*g*_pw_medium_), low (*g*_pw_low_), and dry. Bar, 1 cm. (c) Images of AL with *g*_pw_low_ (top), *g*_pw_medium_ (middle), and *g*_pw_high_ (bottom) under the microscope; bar, 1000 μm. (d) Average pore area (mm^2^) and density (mm^−2^) of the three intermediate AL. Data points show mean ± SE (*n* = 3).

Five AL were built to create DynG: one fully dry and one fully wet AL each for *I*_g_ (Eqn 1) and *g*_bw_ (Eqn 10) calculation, and three AL with fixed, intermediary *g*_pw_ based on their porosity for determination of DynG (*g*_pw_low_ < *g*_pw_medium_ < *g*_pw_high_). All AL were made from Whatman filter paper (No. 1, Cytiva, Marlborough, MA, USA) measuring 2 × 4 cm and with a tail extending *c*. 4 cm from one side (Fig. 2b). The tail was submerged in a water‐filled test tube (except for the dry AL; Supporting Information Fig. S1) to maintain stable *E* during the experiment (for at least 1 h; Fig. S2). The filter paper was attached to a polystyrene plastic plate (for support) using double‐sided carpet tape (HEMA, Amsterdam, the Netherlands; Fig. 2a). The top of the plate was covered with black electrical tape, which had an absorptance of 0.96 in the 400‐ to 700‐nm wavelength range and emissivity of long‐wave radiation (*ε*) of 0.97. In the three intermediate AL, the bottom of the filter paper was covered with a black polyethylene film (*a* = 0.95, *ε* = 0.97). A needle roller (URAQT, Shenzhen, China; needle length = 1 mm) was purchased from Amazon and was used to punch holes into the plastic films (Fig. 2a). The needle roller was rolled across the plastic film either once, twice, or three times to create AL with different pore density (and thus different *g*_pw_). Dry and wet leaves were left uncovered to ensure zero evaporation by dry leaves and unrestricted evaporation by wet leaves. Note that wet leaves were designed to evaporate only from the bottom surface; this approach prevents potential variations in radiation absorption caused by water films forming on the upper surface, as would be the case with wet leaves wetted on both sides (Brissinger *et al*., 2014). The edge of the plastic film was wrapped around the polystyrene plate from all sides (except for the end) and thus created a relatively closed space, ensuring minimal evaporation from nonporous areas. Gloves were worn when making AL to prevent grease on the hands from sticking to the surface and affecting evaporation. As filter paper may degrade and change properties, a set of new AL was prepared before each experiment. AL were found to be highly repeatable in their characteristics, as we found close agreement in pore conductance (measured using gas exchange; Li‐6800 photosynthesis system) between three separate replicates (Fig. S3).

### Measurements

#### Characterization of AL

The porosity of the three intermediate AL was measured with a stereomicroscope (Leica MZ APO MSV269, Leica Microsystems, Wetzlar, Germany), which was connected to a color camera (Axiocam 305 color, Zeiss International, Oberkochen, Germany). The obtained images (Fig. 2c) were processed with imagej (v.1.53, 64‐bit). Average pore area (mm^2^) and density (pores mm^−2^) were calculated based on 8 to 16 pictures (Fig. 2d). From these measurements, the theoretical anatomical maximum *g*_pw_ (mol m^−2^ s^−1^) of each plastic film was estimated following Dow *et al*. (2014) (Table 1):

(Eqn 8) $$g_{\mathrm{pw}}=\frac{\mathrm{PD}\times D\times \mathrm{PA}}{V\times \left(l+n\times \left(\sqrt{\frac{\mathrm{PA}}{\pi }}\right)\right)}$$

where PD is pore density (pores m^−2^), *D* is the diffusivity of water in the air (m^2^ s^−1^), PA is the average pore area (m^2^ pore^−1^), *V* is the molar volume of air (m^3^ mol^−1^), *l* is the thickness of the plastic film (m), and *n* is an end correction factor (see Table 2, for symbol description and units). In AL, water vapor forms ‘vapor shells’ at each pore, which accumulate near the pore outlet and may interfere with each other, resulting in longer or more complex vapor diffusion paths, thus adding diffusion resistance (Lehmann & Or, 2015). This phenomenon is known as end correction resistance (*R*_end_).

**Table 1:** Comparison between theoretical maximum *g*_pw_ based on porosity and measured *g*_pw_ using gas exchange.

Correction factor	Theoretical anatomical maximum *g*_pw_ (mol m^−2^ s^−1^)			*g*_pw_ measured by gas exchange (mol m^−2^ s^−1^)
	*π*/4	*π*/2	1	
*g*_pw_low_	0.09	0.06	0.08	0.10 ± 0.007^c^
*g*_pw_medium_	0.19	0.11	0.16	0.20 ± 0.006^b^
*g*_pw_high_	0.27	0.16	0.23	0.29 ± 0.013^a^

Several correction factors (*n*) were considered to account for additional diffusive resistance. Values by gas exchange measurements are mean ± SE, *n* = 3. Different letters indicate significant differences as determined by Tukey's test (*P* < 0.05).

**Table 2:** Abbreviation and parameters list.

Symbol	Description	Unit
AL	Artificial leaves	
*C*_p_	Specific heat capacity of humid air	J kg^−1^ K^−1^
*D*	Diffusivity of water vapor in the air	m^2^ s^−1^
DynG	Slope of the *I*_g_‐ *g*_pw_ relationship	dimensionless
*e*_*s*_, *e*_*a*_	Leaf internal (*e*_s_) and air (*e*_a_) vapor pressure	Pa
*E*	Transpiration rate	g m^−2^ s^−1^
*g*_bw_	Boundary layer conductance to water vapor	mol m^−2^ s^−1^
*g*_HR_	Boundary layer conductance to heat and radiative transfer	mol m^−2^ s^−1^
*g*_pw_	Pore conductance to water vapor	mol m^−2^ s^−1^
*g*_R_	Boundary layer conductance to radiative transfer	mol m^−2^ s^−1^
*g*_sw_	The sum of cuticular and stomatal conductances to water vapor	mol m^−2^ s^−1^
*g*_tw_	Total leaf conductance to water vapor	mol m^−2^ s^−1^
*G*	Slope of the *I*_g_‐ *g*_sw_ relationship	dimensionless
*I*_g_	Conductance index	dimensionless
*l*	Thickness of plastic film	m
*n*	End correction factor	dimensionless
*P*_atm_	Atmospheric pressure	Pa
PA	Average pore area	m^2^ pore^−1^
PD	Pore density	pores mm^−2^
PPFD	Photosynthetic photon flux density	μmol m^−2^ s^−1^
*R*	Gas constant	m^3^ Pa K^−1^ mol^−1^
RH_air_	Air relative humidity	dimensionless
*R*_*ni*_	Net isothermal radiation	W m^−2^
*s*	Slope of the curve relating saturation water vapor pressure to *T*_air_	Pa K^−1^
SR	Ratio of stomatal densities between the ad‐ and abaxial leaf sides	dimensionless
*T*_air_	Air temperature	K
*T*_dry_	Temperature of a dry (non‐transpiring) reference	K
*T*_leaf_	Leaf temperature	K
*T*_wet_	Temperature of a wet (fully transpiring) reference	K
*V*	Molar volume of air	m^3^ mol^−1^
VPD	Air vapor pressure deficit	Pa
*α*	Leaf absorptance of short‐wave radiation (400–700 nm)	dimensionless
*ℇ*_*l*_, *ℇ*_*B*_, *ℇ*_*F*_	Long‐wave radiation emissivity of leaf, black electrical tape and filter paper	dimensionless
*ρ*_air_	Density of air	kg m^−3^
*γ*	Psychrometric constant	Pa K^−1^
*λ*	Latent heat of evaporation of water	J kg^−1^
*σ*	Stefan–Boltzmann constant	W m^−2^ K^−4^

A distance may exist between the pores in the plastic film and the transpiring surface of filter paper, which affects the degree of interaction of the vapor shells and the diffusion path of water vapor. Therefore, correction factors such as one‐end (*n* = *π*/4), two‐end (*n* = *π*/2), or no correction (*n* = 1) were considered to quantify the additional diffusive resistance. The one‐end and two‐end correction factors account for vapor shells formed only at the outer ends or both ends of the stomatal cavity, respectively, while no correction represents an intermediate value.

#### Experimental setup

Measurements were conducted in an enclosure covered with black light‐proof fabric (210 × 120 × 75 cm) to maintain a stable environment, which was located in a temperature‐controlled laboratory with *T*_air_ of 20°C (Zhang *et al*., 2024). An infrared thermal camera (FLIR A655sc; FLIR system, Inc., Wilsonville, OR, USA) with an uncooled microbolometer detector (resolution: 640 × 480 pixels; spectral range: 7.5–14.0 μm; noise equivalent temperature difference: <30 mK) was mounted on a tripod and placed on the left side of the enclosure. Temperature changes of real and ALs were continuously captured using the researchir max software (FLIR, v.4.40.12.38). Software settings included the distance between the camera and objects (0.6 m), atmospheric conditions (*T*_air_ and RH_air_), and reflected temperature (temperature of a crumpled sheet of aluminum foil near the target object, set to *ε* = 1; (Usamentiaga *et al*., 2014)). *T*_air_ and RH_air_ were monitored every 0.1 s by a sensor (SHT‐45; Sensirion, Stafa, Switzerland) connected to a digital reader (SEK‐SensorBridge; Sensirion). An electrical fan was placed next to the thermal camera to ensure air mixing, and wind speed was measured using an anemometer (Voltcraft PL‐135 HAN; Conrad Electronics, Hirschau, Germany). A LED light source (Elixia; Heliospectra AB, Göteborg, Sweden) containing a blue (peak: 450 nm), red (660 nm), and white LED channel, located on the right side of the enclosure, provided illumination. Leaf light absorptance of Arabidopsis genotypes was measured with a spectrophotometer connected to an integrating sphere and was determined to be WT: 0.84 ± 0.004 (mean ± SEM, *n* = 3), EPF: 0.84 ± 0.003, and *epf1epf2*: 0.85 ± 0.006, respectively. No significant differences between genotypes were detected (*P* = 0.68).

### Experimental design

#### Testing the performance of *I*_g_ and DynG

Five reference AL and another target AL were placed under three wind speeds (0.1, 0.4, 0.9 m s^−1^; in darkness) and four light intensities (0, 100, 300, 500 μmol m^−2^ s^−1^). The target AL was created by the same procedure as the ‘High’ reference AL (‘Construction of AL’ in the Materials and Methods section); its *g*_pw_ value remains constant regardless of changes in the surrounding environment, thereby serving as a benchmark to test the performance of *I*_g_ and DynG on *g*_pw_ estimation under dynamic conditions. The distance between the fan and AL was modified to ensure that all AL experienced similar wind speeds. A LI‐180 spectrometer (LI‐COR, Lincoln, NE, USA) was used to ensure homogeneous light intensity near AL. Temperature kinetics of AL were recorded, and *I*_g_ of intermediate AL was calculated using Eqn 1. The target AL was measured using a gas exchange system (Li‐6800). The gas exchange cuvette was set to two different sets of environmental conditions to provide *g*_pw_ benchmark data in wind speed and light intensity tests: The first set was 300 μmol s^−1^ air flow rate, 21°C *T*_air_, 40% RH_air_, and 5‐min darkness for wind speed test (*g*_pw_ = 0.31 ± 0.012 mol m^−2^ s^−1^). The second set of conditions was 300 μmol s^−1^ air flow rate, 40% RH_air_, and four light intensities (0, 100, 300, 500 μmol m^−2^ s^−1^, each lasting 3 min), and leaf temperatures of 19.5, 20.0, 20.7, and 21.8°C, corresponding to AL temperatures in the thermal imaging setup under the same light intensities. *g*_pw_ was measured three times by inserting the target AL three separate times.

#### Distinguishing genotypes with different stomatal density

DynG was tested on the three Arabidopsis genotypes. Studies suggest that SR in Arabidopsis is *c*. 0.8 to 1 (McAusland *et al*., 2016; Jalakas *et al*., 2024; Tulva *et al*., 2024); so, for simplification, we assumed SR = 1. This assumption satisfies the conditions for using Eqn 7 to obtain *g*_sw_. One replicate per genotype and five AL were placed side by side under the LED lamp (Fig. S4a). Since Arabidopsis leaves had a lower light absorptance (0.84) than AL (0.96), plants were placed *c*. 2 cm closer to the light source than AL, and a LI‐180 spectrometer was used to ensure identical light intensity between AL and plants. Wind speeds (0.4 m s^−1^) around plants and AL were homogenized by adjusting their positions relative to the fan. Objects were then subjected to 0, 150, and 300 μmol m^−2^ s^−1^ for > 1.5 h per photosynthetic photon flux density (PPFD) to ensure stable *g*_sw_. Five nonoverlapping leaves per plant were selected for *T*_leaf_ recordings; target areas on these leaves were outside of the pot's diameter to prevent interference with the temperature of the soil and the film cover above the pot. The average temperature of all selected leaf area was used to represent whole‐rosette temperature, and 25 thermograms were recorded per second (25 Hz). *I*_g_ of AL was determined using Eqn 1, and *g*_sw_ of Arabidopsis plants was estimated by inputting the obtained *I*_g_ into the *I*_g_ *f*(*g*_pw_) function.

### Method validation

#### Validation of *g*_pw_ with gas exchange measurements

Calculated *g*_pw_ was validated using a gas exchange system (LI‐6800) equipped with a 2‐cm^2^ Chl fluorometer cuvette. Each AL with known *g*_pw_ was created in triplicate. Before measurements, the tails of AL were immersed in water‐filled tubes for 1 h in the laboratory, at 20°C *T*_air_ and 40 ± 5% RH_air_. The bottom evaporating surface of the AL was squeezed vigorously using dry tissues to ensure that no droplets adhered to it. Environmental conditions in the gas exchange cuvette were set to 300 μmol s^−1^ air flow rate, 21°C T_air_, 40% RH_air_, and darkness. *G*_pw_ was measured after 5 min of stabilization (Fig. S3).

#### Comparison between DynG and lysimetric method

Changes in plant weight due to *E* were measured to verify DynG. To prevent evaporation from the substrate, pots were wrapped in black plastic film. Col‐0 plants adapted to different light intensities (0, 150, and 300 μmol m^−2^ s^−1^; > 1.5 h per PPFD) were placed on a precision scale (FPRS822; Thermo Fisher Scientific Inc, Leicestershire, UK) in the same setup, which automatically recorded the weights per second, and were subjected to either 0, 150, or 300 μmol m^−2^ s^−1^ for 20 min (Fig. S4b). The thermal camera captured leaf and AL temperature to determine *I*_g_. Leaves were removed from the pot, placed on a whiteboard next to a ruler, photographed, and leaf area per plant was estimated using imagej (v.1.53, 64‐bit). *E* (in g m^−2^ s^−1^) was calculated by estimating the slope of weight loss per unit time, divided by leaf area. *g*_sw_ was obtained as described previously (see the ‘Comparison between DynG and lysimetric method’ section) and was used to calculate *E* as modified from Vialet‐Chabrand & Lawson (2020):

(Eqn 9) $$\;E=0.018\times \frac{g_{\mathrm{bw}\times }\;g_{\mathrm{sw}}}{g_{\mathrm{bw}}+g_{\mathrm{sw}}}\times \left(\frac{e_{s}-e_{a}}{P_{\mathrm{atm}}}\right)$$

where 0.018 is the molecular weight of water (kg mol^−1^), *e*_*s*_ is the leaf internal vapor pressure (Pa) at saturation, and *e*_*a*_ is the air vapor pressure (Pa).

We additionally applied the lysimetric method to calculate *g*_sw_ using *E* as input, and compared these *g*_sw_ values with those obtained through the DynG method, using Col‐0 (*n* = 3). Furthermore, we compared the *g*_sw_ calculated by *I*_g_ (*g*_sw_ = *I*_g_ as used in Pignon *et al*., 2021) with the results of the lysimetric method.

#### Estimation and validation of *g*_bw_

Based on the steady‐state leaf energy balance, the equation proposed by Vialet‐Chabrand & Lawson (2020) was modified to calculate *g*_bw_ by using dry and wet leaves:

(Eqn 10) $$g_{\mathrm{bw}}=\frac{T_{\mathrm{dry}}-T_{\mathrm{wet}}-\sigma \times \left({T_{\mathrm{wet}}}^{4}-{T_{\mathrm{dry}}}^{4}\right)\times \left(\varepsilon_{B}+\varepsilon_{F}\right)}{\lambda \times 0.018\times \left(\frac{e_{s}-e_{a}}{P_{\mathrm{atm}}}\right)+2\times \rho_{\mathrm{air}}\times C_{p}\times \left(\frac{0.92\times R\times T_{\mathrm{wet}}}{P_{\mathrm{atm}}}\right)\times \left(T_{\mathrm{dry}}-T_{\mathrm{wet}}\right)}$$

where *ε*_*B*_ is the emissivity of the upper surface of AL (0.97 for black electrical tape), *ε*_*F*_ is the emissivity of the lower surface of AL (0.95 for filter paper), *λ* is the latent heat of evaporation of water (J kg^−1^), (0.92 × *R* × *T*_wet_)/*P*_atm_ is a conversion factor for *g*_bw_ from heat transfer (m s^−1^) to gas exchange (mol m^−2^ s^−1^), where *R* is the gas constant (8.3145 J mol^−1^ K^−1^). A sensitivity analysis was performed to characterize the extent to which changes in *g*_bw_ (± 50%) and SR (0.5 < *K* < 1.5) in Eqn 7 affect *g*_sw_.

To further verify *g*_bw_ obtained through Eqn 10, a comparison was made with *g*_bw_ values derived using the lysimetric method. Dry and wet references were placed on the precision scale for 0.5 h in the dark (Fig. S5), and the change in weight was recorded (no change in weight was noticed in the dry reference). As *g*_sw_ was considered infinite in the wet leaf, Eqn 10 was rewritten as:

(Eqn 11) $$g_{\mathrm{bw}}=\frac{E}{0.018\times \left(\frac{e_{s}-e_{a}}{P_{\mathrm{atm}}}\right)}$$

This experiment was repeated three times, with new dry and wet ALs being made each time.

### Data analysis

Eqns (Eqn 2), (Eqn 3), (Eqn 4), (Eqn 5), (Eqn 6), (Eqn 7), (Eqn 8), (Eqn 9), (Eqn 10), (Eqn 11) were implemented in R (R project, v.4.2.0). For mean testing, the normality of residuals was tested using a Shapiro–Wilk test. A one‐way ANOVA was performed to test for significant differences between Arabidopsis genotypes, and a Tukey's Honestly Significant Difference (HSD) *post hoc* test was used for mean separation. Statistics were made in R using the aov() and TukeyHSD() functions. A Student's *t*‐test was used to compare paired samples (dry and wet references) in the *g*_bw_ validation. All codes are available on GitHub (https://github.com/jiayu0903/dynamic‐conductance‐index.git). A spreadsheet containing an example of calculating *g*_bw_ (Eqn 10), *g*_sw_ (Eqn 7) and *E* (Eqn 9) based on DynG and microclimatic parameters is also provided in Dataset S1.

## Results

### *g*_pw_ of intermediate artificial leaves (ALs)

The *g*_pw_ of the three intermediate AL that differed in porosity followed the expected trend, namely: *g*_pw_high_ > *g*_pw_medium_ > *g*_pw_low_ (Table 1; *P* < 0.05). This trend was evident both for calculated values based on measured porosity and from gas exchange measurements on AL. Accounting for the water vapor shell around the pores, we further found that using the one‐end correction factor (*n* = *π*/4), values of calculated *g*_pw_ were very close to measured *g*_pw_ (Table 1). By contrast, we found that using no (*n* = 1) or a two‐end correction factor (*n* = *π*/2) would underestimate true *g*_pw_.

### *I*_g_ and DynG under dynamic environmental conditions

**Fig. 3:**
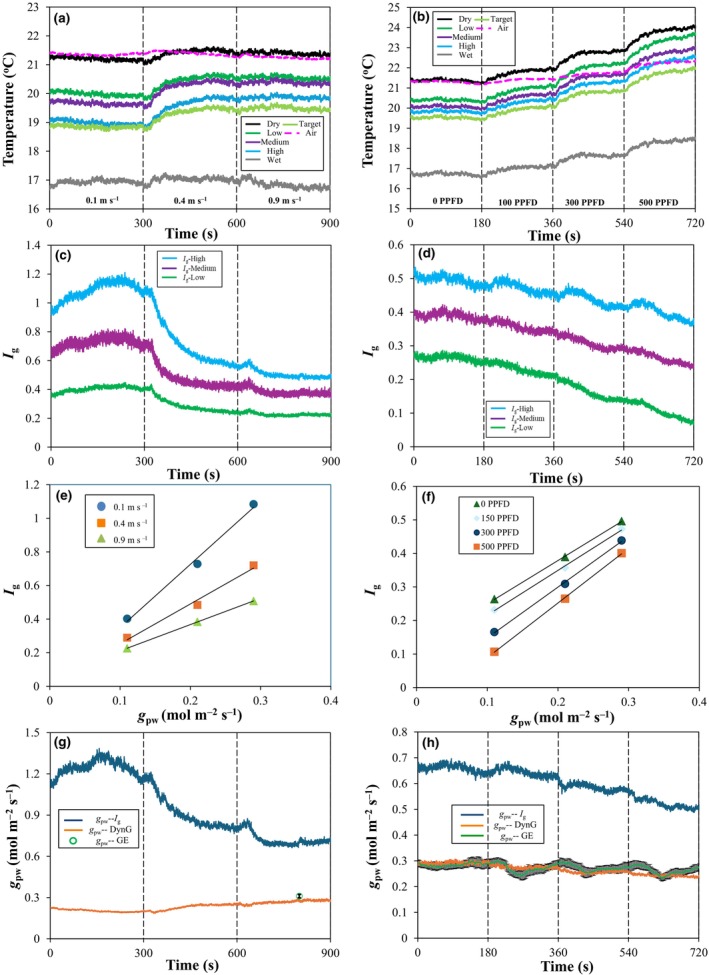
DynG accurately captures the pore conductance (*g*_pw_) of an artificial leaf (AL) with known porosity, whereas *I*_g_ does not. (a,b) temperature of AL (dry, low, medium, high, wet, and target) and air (*T*_air_). (c,d) *I*_g_ kinetics of three intermediate AL. (e,f) relationships between *I*_g_ and *g*_pw_ of three intermediate AL (Table 1). (g,h) comparison between *g*_pw_ as inferred using *I*_g_ (*g*_pw_–*I*_g_; Eqn 1) or DynG (*g*_pw_‐DynG; Eqn 7), or as measured using gas exchange (*g*_pw_‐GE; means ± SE, *n* = 3) on the target AL. The left column shows effects of wind speed (0.1, 0.4, and 0.9 m s^−1^), while the right column shows effects of PPFD (0, 100, 300, and 500 μmol m^−2^ s^−1^). Vertical dotted lines show times at which a change in these factors occurred.

We tested the performance of DynG by exposing five AL and one target AL to a range of wind speeds and light intensities. Except for the target AL, the temperature kinetics of intermediate AL consistently stayed within the limits set by wet and dry references, with the ‘High’ AL achieving the most and ‘Low’ the least cooling, respectively (Fig. 3a,b). As wind speed increased, the temperature of all AL gradually increased (Fig. 3a; except for the dry and wet reference, which showed a small reduction under high wind speed). The temperature difference between dry and wet references showed a similar trend, increasing with wind speed and light intensity (Fig. S6a,b). Temperatures of air (*T*_air_) and of the dry reference were nearly identical under different wind speeds (Fig. 3a), but diverged under different light intensities, as the dry reference heated up with increasing PPFD (Fig. 3b).

Although *g*_pw_ of a given AL is constant, *I*_g_ reduced with increases in either wind speed or PPFD (Fig. 3c,d). Using the three intermediate AL, we found that the slope of the *I*_g_ vs *g*_pw_ relationship (DynG) was highly linear and decreased when wind speed and PPFD increased (Fig. 3e,f). The *g*_pw_ of target AL as inferred from *I*_g_ (*g*_pw_–*I*_g_; Eqn 1) showed reductions as wind speed or light intensity increased (Fig. 3g,h). By contrast, *g*_pw_ as inferred from DynG (*g*_pw_‐DynG; Eqn 7) was comparably stable and closely aligned with the value of *g*_pw_ as measured using gas exchange (*g*_pw_‐GE; Fig. 3g,h). We also observed that as PPFD increased, both *g*_pw_‐DynG and *g*_pw_‐GE showed a slight decrease (Fig. 3h).

### DynG validation and sensitivity analysis

**Fig. 4:**
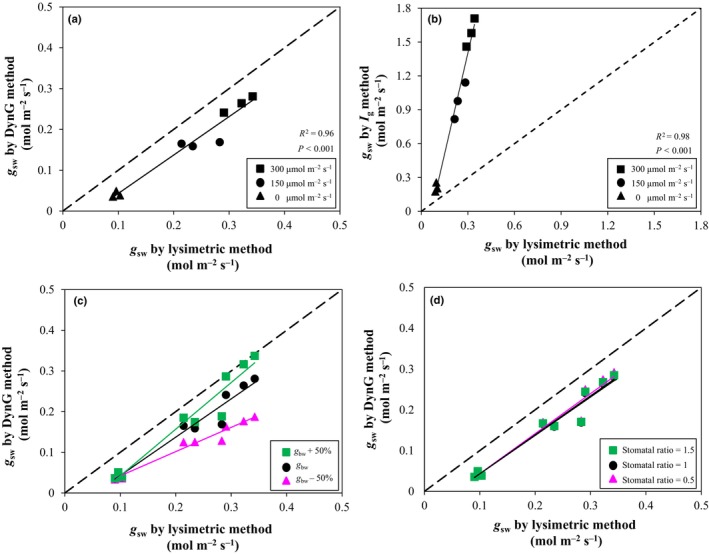
Validation of DynG by the lysimetric method, and sensitivity analysis of key parameters. Comparison of stomatal conductance to water vapor (*g*_sw_) of Arabidopsis Col‐0 by using lysimetric between (a) DynG methods (Eqn 7) and (b) *I*_g_ methods (Eqn 1) at different PPFD and low wind speed (*c.* 0.2 m s^−1^). Sensitivity analysis of *g*_sw_ by DynG when (c) boundary layer conductance to water vapor (*g*_bw_) changed by ±50% and (d) stomatal ratio (SR) changed from 0.5 to 1.5. Note that single‐replicate values are displayed.

Whole‐plant E of Col‐0 plants was determined by either using DynG (Eqn 7) or through the rate of weight loss (Fig. S7). Before estimating *E*, *g*_bw_ of the dry and wet reference (Eqn 10) was validated by tracking weight loss of the wet reference, and no significant difference between methods was found (Fig. S5). Whole‐plant *E* or *g*_sw_ as determined by either DynG or the lysimetric method showed strong positive linear correlations (*R* ^2^ = 0.96–0.97), but with a consistent bias toward the lysimetric method. Additionally, values of *g*_sw_ as calculated using *I*_g_ strongly deviated from the 1 : 1 line when compared with the lysimetric method (Fig. 4a,b), confirming that DynG can provide much more accurate estimates of *g*_sw_ than *I*_g_.

To detect the extent to which changes in leaf boundary layer (*g*_bw_) and SR affect the estimation of *g*_sw_, a sensitivity analysis was conducted. We found that a +50% change in *g*_bw_ resulted in a 3 to 20% change in simulated *g*_sw_, whereas when *g*_bw_ changed by −50%, the change in *g*_sw_ was more pronounced, ranging from 7 to 33%, with both effects increasing as light intensity rose (Fig. 4c). On the other hand, different SR (from 0.5 to 1.5) had minimal effects on *g*_sw_ (Fig. 4d).

### Using DynG to distinguish *g*_sw_ between genotypes

**Fig. 5:**
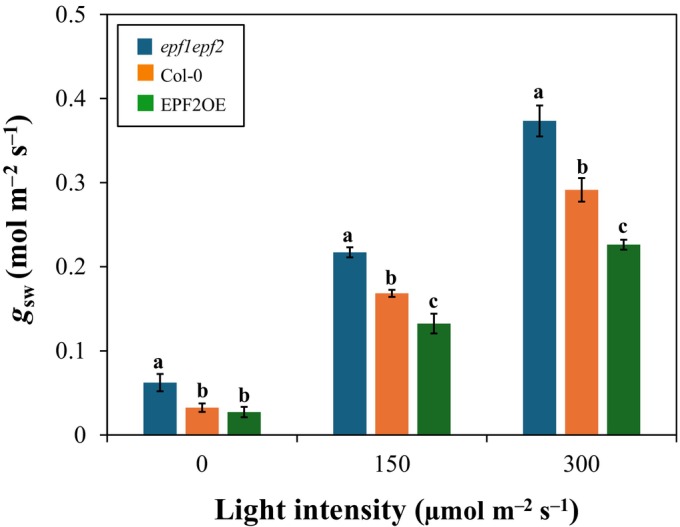
DynG can be used to distinguish stomatal conductance (*g*_sw_) between Arabidopsis genotypes at different light intensities. Different letters signify significantly different (*P* < 0.05) values according to Tukey's HSD test; bars represent the SE of the means (*n* = 3).

We tested whether DynG could detect differences in *g*_sw_ across three Arabidopsis genotypes with varying stomatal density and under different light intensities. All genotypes showed increased *g*_sw_ when PPFD was increased (Fig. 5). Also, at each PPFD, *g*_sw_ differed significantly between genotypes (except for Col‐0 and EPF2OE in darkness), and these differences became larger as PPFD increased. This suggested that *g*_sw_ as derived from DynG can be used to distinguish different genotypes, especially in plants adapted to higher PPFD.

## Discussion

### A novel method to estimate stomatal conductance

Measuring stomatal conductance (*g*_sw_) from thermography has been notoriously difficult, especially in a quickly changing environment. To rapidly and accurately estimate *g*_sw_ regardless of environmental changes, we propose here a dynamic scaling factor (termed ‘DynG’) using easy‐to‐make leaf replicas. The use of DynG allowed for more accurate predictions of *g*_pw_ and *g*_sw_ compared with *I*_g_ (Figs 3g,h, 4a,b) in dynamic environments, opening new avenues for monitoring stomatal behavior as derived from thermography‐based techniques. Whole‐plant *E* of WT Arabidopsis (Col‐0) estimated from weight loss correlated very well with *E* as derived from DynG (Fig. 4a), and further tests demonstrated that DynG successfully distinguished *g*_sw_ of different Arabidopsis genotypes differing in stomatal density (Fig. 5), suggesting that DynG can be a useful proxy for *g*_sw_ estimation in HTP.

### DynG predicted *g*_sw_ accurately under different microclimates

A change in *g*_bw_ due to variations in wind speed is known to affect *I*_g_ (Jones, 1999a), which we also observed (Fig. 3c). *g*_bw_ normally increases with wind speed, and similar results were found in Fig. S6c. It was previously reported that *I*_g_ was unaffected by incoming radiation (Maes & Steppe, 2012). By contrast, we found that *I*_g_ decreased when the light intensity increased (Fig. 3d). This difference in results may be attributed to light‐induced microclimate variations affecting the stability of *I*_g_. Although *g*_bw_ was relatively constant, the increased air temperature and reduced air humidity together increased VPD of air, and *G* is sensitive to VPD. The latent cooling capacity of the AL also increased, amplifying the difference between *T*_dry_ and *T*_wet_ (Fig. S6b), and leading to lower *I*_g_.

*I*_g_ of individual AL changed with the ambient microclimate, while DynG is based on the temperature kinetics of dry and wet AL, plus three AL with different and known pore conductance (*g*_pw_). These AL can track environmental changes and thus provide *I*_g_–*f* (*g*_pw_) at any moment to determine *g*_sw_ of leaves in the field of view of the thermal camera, representing an improvement compared with *I*_g_, which only uses dry and wet leaves (Pignon *et al*., 2021; Savvides *et al*., 2022).

Unlike in real leaves in which pore width can change as stomata open or close, any AL should have a relatively fixed *g*_pw_ with unchanging pore width. Consequently, *g*_pw_ estimated from different methods should remain stable when the microclimate changes, which was supported by our observations. We found that *g*_pw_ based on DynG (*g*_pw_‐DynG) was relatively stable and close to values determined using gas exchange under a range of PPFD and wind speeds (*g*_pw_‐GE). However, this was not the case for *g*_pw_ based on *I*_g_ (*g*_pw_–*I*_g_), which was highly variable (Fig. 3g,h), highlighting the benefit of using DynG under dynamic environments. While DynG demonstrated a robust performance under fluctuating conditions (e.g. wind, light), further testing using mutants that are insensitive to environmental stimuli (e.g. *ost1* under different VPD; Merilo *et al*., 2018) would be informative and valuable for future research.

The *g*_sw_ calculated from DynG was much closer to values obtained from the lysimetric method than when calculated from *I*_g_ (Fig. 4b) and showed a more stable trend (Fig. 5b). Arabidopsis leaves normally have stomata on both sides (Pillitteri & Dong, 2013), while AL transpire from only one side, leading to differences in energy balance. The *g*_sw_ of real leaves is not simply proportional to the *I*_g_ of AL, and we therefore considered the stomatal distribution and variations in *g*_bw_ (Guilioni *et al*., 2008). Interestingly, our sensitivity analysis (Fig. 5d) showed that changes in stomatal ratio (0.5 < *K* < 1.5) hardly affected calculated *g*_sw_, suggesting that for amphistomatous leaves, using *K* = 1 can simplify the calculations and provide sufficiently accurate results. By contrast, variations in *g*_bw_ clearly affected calculated *g*_sw_ (Eqn 7; Fig. 5c), suggesting that accurate estimation of the factors driving g_bw_ is crucial (e.g. wind speed nearby the AL).

The *g*_sw_ values as estimated through DynG were always slightly lower than those obtained using the lysimetric method (Fig. 5b). This may be due to *g*_bw_ of AL not fully reflecting that of real leaves. More accurate values of *g*_sw_ under high‐light intensities can be obtained by increasing *g*_bw_ by 50% (Fig. 4c). We calculated *g*_bw_ based on the difference in steady‐state energy balance between a dry and wet AL (Eqn 10), which could be considered an improvement compared with previous methods that required additional energy input, either in the form of heated leaf replicas or through cooling profiles when *T*_leaf_ was raised above *T*_air_ (Brenner & Jarvis, 1995; Katsoulas *et al*., 2006; Stokes *et al*., 2006; Kimura *et al*., 2016; Kimura *et al*., 2020). Although the *g*_bw_ of AL can be estimated by the dry and wet reference method accurately (Fig. S5), the boundary layer conditions may be different in real leaves compared with AL because of differences in leaf shape, dimensions, presence of trichomes, and edge effects over the lamina (Defraeye *et al*., 2013; Schymanski & Or, 2016; Kimura *et al*., 2020). DynG can further quantify the *g*_sw_ variations of different Arabidopsis genotypes under step changes in light intensity (Fig. 5). The range of *g*_sw_ obtained from these genotypes was similar to those described in previous studies (Doheny‐Adams *et al*., 2012; Driever *et al*., 2023), suggesting that DynG can provide comparable and physiologically meaningful results. Notably, the *epf1epf2* double mutant showed significantly higher *g*_sw_, consistent with its increased stomatal density (*c*. 225% higher) and clustering compared with Col‐0 (Hunt & Gray, 2009; Doheny‐Adams *et al*., 2012), while EPF2OE exhibited reduced *g*_sw_, consistent with an *c*. 80% decrease in stomatal density (Hara *et al*., 2009; Franks *et al*., 2015). This agreement with established physiological differences supports the applicability of DynG in resolving genotype‐specific stomatal responses.

### Possible applications of DynG

HTP is widely used in plant research and breeding to quantify phenotypic traits across diverse plant species, from model organisms such as Arabidopsis to major crops including wheat, maize, and rice (Yang *et al*., 2013; Araus & Cairns, 2014; Arend *et al*., 2016). A bottleneck in HTP related to plant gas exchange is uneven air mixing (Boulard *et al*., 2004; Katsoulas *et al*., 2006), such that results at different times or locations on the same platform may be inconsistent, thereby affecting the accuracy and comparability of phenotyping data. Uneven air mixing affects *g*_bw_, which in turn affects *E* and *g*_sw_ (Monteith, 1995). A typical future application of thermography in HTP could be to place a set of AL in an imaging box on a plant‐to‐sensor platform (i.e. platforms in which plants are moved along fixed phenotyping stations), and utilize DynG for *g*_sw_ monitoring of each plant entering the box, while providing comparable results that account for the effects of unstable microclimates.

In modern protected cultivation systems such as vertical farms and glasshouses, crop monitoring is essential for improving yields and/or energy use efficiency (van Delden *et al*., 2021; Steeneken *et al*., 2023; Kaiser *et al*., 2024), particularly for key physiological traits such as gas exchange (Kläring & Körner, 2020). Plant–environment interactions are complex, and understanding the plant's real‐time status – which affects its light and water use efficiencies – can significantly aid growers' decision‐making. While experienced growers rely on environmental data to predict plant status, this approach is typically limited to long‐term trends and fails to capture short‐term (e.g. diurnal) or localized changes (Kaiser *et al*., 2024). This highlights the need for robust and precise sensors to track plant traits over time, and for simple methods to interpret the signals derived from said sensors. Our AL ‐based DynG offers a way to conduct continuous *g*_sw_ monitoring of crops in their production environment. Furthermore, dry and wet AL may be used for a different kind of sensing: by monitoring temperature changes of AL to calculate *g*_bw_ in real time and feeding back to the ventilation system, airflow can be intelligently optimized to save electricity (Davies *et al*., 2008).

### Points of attention and limitations

Accurately estimating *g*_pw_ of AL is a prerequisite for applying DynG. While *g*_pw_ of a given AL obviously depends on pore size and density, nonlinear diffusion due to the formation of vapor shells may introduce additional diffusion resistance (Lehmann & Or, 2015), which can be corrected by using appropriate ‘end correction’ factors. We found that in our AL, *g*_pw_ as measured using gas exchange closely matched calculated theoretical maximum anatomical *g*_pw_, as long as the latter used an end correction factor of ‘*π*/4’ (Table 1). This suggests that the AL used by us had a relatively tight structure, effectively reducing the diffusion path of water vapor (Franks & Farquhar, 2001). Additionally, good airflow is necessary, as it affects gas diffusion rate near the surface: Under low wind speed (0.1 ms^−1^), a buildup of humidity (Fig. S6) can result in reduced VPD and impose a temporary reduction on evaporative demand (Grange & Hand, 1987; Fanourakis *et al*., 2016), thereby affecting *g*_pw_.

For plants with a more complex morphology than Arabidopsis, the application of *I*_g_ and DynG is both limited by heterogeneity in light interception throughout the plant as caused by different leaf angles and orientations (Zhang *et al*., 2024), as well as the different microenvironments (including air mixing) around the leaves. The use of a set of AL placed at different angles may be a solution for differences in energy balance between such leaves. In addition, absorbed short‐wave radiation accounts for a significant portion of the leaf's energy balance, particularly in the presence of external light input. DynG assumes that real and ALs absorb light equivalently; follow‐up studies could select materials with optical properties similar to those of the target crop to match their leaf absorptance, simplifying the experimental procedure. Additionally, recent research has replicated the microstructure of leaves using soft lithography (Soffe *et al*., 2019; Bohlim *et al*., 2021); this could be used to design more realistic AL. In this study, a 'tail' attached to each AL was immersed in water to ensure continuous water supply, allowing the AL to maintain a stable temperature for > 1 h (Fig. S2). To extend the usage time, the AL could be connected to a liquid flow meter or reservoir, as was done in previous studies (Grant *et al*., 2016; Maes *et al*., 2016; Schymanski *et al*., 2017), which can provide a more continuous water supply. However, these designs are more complex and bulkier.

### Conclusions

Our novel method provides an easy correction factor ‘DynG’ that can be applied to estimate accurately leaf *g*_sw_ from thermal imaging with a set of low‐cost leaf replicates under fluctuating environmental conditions. By using DynG to correct the conductance index (*I*_g_) and calculate *g*_sw_ values, we successfully phenotyped different Arabidopsis genotypes without the bias generally observed with *I*_g_. This enables the rapid phenotyping or monitoring of *g*_sw_ and *g*_bw_, and provides more comparable and reliable data under different environmental conditions compared with the earlier index, *I*_g_.

## Competing interests

None declared.

## Author contributions

SV‐C, EK and JZ conceived and designed the experiments. JZ performed experiments and analyzed data with the help of SV‐C. JZ, EK, LM and SVC wrote the manuscript. All authors commented and approved the manuscript.

## Disclaimer

The New Phytologist Foundation remains neutral with regard to jurisdictional claims in maps and in any institutional affiliations.

## Supporting information


**Dataset S1** Spreadsheet for calculating *g*_sw_ based on DynG.

**Fig. S1** An overview of the artificial leaves (ALs), including the materials, tools, and perspectives from various angles.
**Fig. S2** Temperature kinetics of five artificial leaves (wet, dry, high, medium, and low), as well as air temperature (*T*_air_), when left in the dark for > 1 h.
**Fig. S3** Time courses of pore conductance to water vapor (*g*_pw_) of three artificial leaves (ALs) in the dark, as determined by gas exchange (Li‐6800). (a) Low *g*_pw_, (b) Medium *g*_pw_, and (c) High *g*_pw_. Each line represents a different replicate.
**Fig. S4** Overview of experimental setup for distinguishing different genotypes, and DynG validation by the lysimetric method.
**Fig. S5** Validation of method to determine boundary layer conductance to water vapor (*g*_bw_) in AL.
**Fig. S6** Factors underlying DynG under environmental changes.
**Fig. S7** Comparison of transpiration rate (*E*) of Arabidopsis Col‐0 by using lysimetric and DynG methods (Eqn 7) at low wind speed (*c.* 0.2 m s^−1^).
Please note: Wiley is not responsible for the content or functionality of any Supporting Information supplied by the authors. Any queries (other than missing material) should be directed to the *New Phytologist* Central Office.

## Data Availability

Related codes are available on GitHub (https://github.com/jiayu0903/dynamic‐conductance‐index.git).
