# Supplementary material for: DynG: a dynamic scaling factor for thermographic stomatal conductance estimation under changing environmental conditions
Source: New Phytol. 2025 Sep 6;248(4):2160–73. doi: 10.1111/nph.70555 (PMC12529036; doi:10.1111/nph.70555)
Supplement: Supplementary file 2 — Fig. S1 An overview of the artificial leaves (ALs), including the materials, tools, and perspectives from various angles. Fig. S2 Temperature kinetics of five artificial leaves (wet, dry, high, medium, and low), as well as air temperature (T air), when left in the dark for > 1 h. Fig. S3 Time courses of pore conductance to water vapor (g pw) of three artificial leaves (ALs) in the dark, as determined by gas exchange (Li‐6800). (a) Low g pw, (b) Medium g pw, and (c) High g pw. Each line represents a different replicate. Fig. S4 Overview of experimental setup for distinguishing different genotypes, and DynG validation by the lysimetric method. Fig. S5 Validation of method to determine boundary layer conductance to water vapor (g bw) in AL. Fig. S6 Factors underlying DynG under environmental changes. Fig. S7 Comparison of transpiration rate (E) of Arabidopsis Col‐0 by using lysimetric and DynG methods (Eqn 7) at low wind speed (c. 0.2 m s−1). Please note: Wiley is not responsible for the content or functionality of any Supporting Information supplied by the authors. Any queries (other than missing material) should be directed to the New Phytologist Central Office. [file NPH-248-2160-s001.docx]

New Phytologist Supporting Information

Article title: DynG: A dynamic scaling factor for thermographic stomatal conductance estimation under changing environmental conditions.

Authors: Jiayu Zhang, Elias Kaiser, Leo F.M Marcelis, Silvere Vialet-Chabrand*.

Article acceptance date: 15 August 2025

**
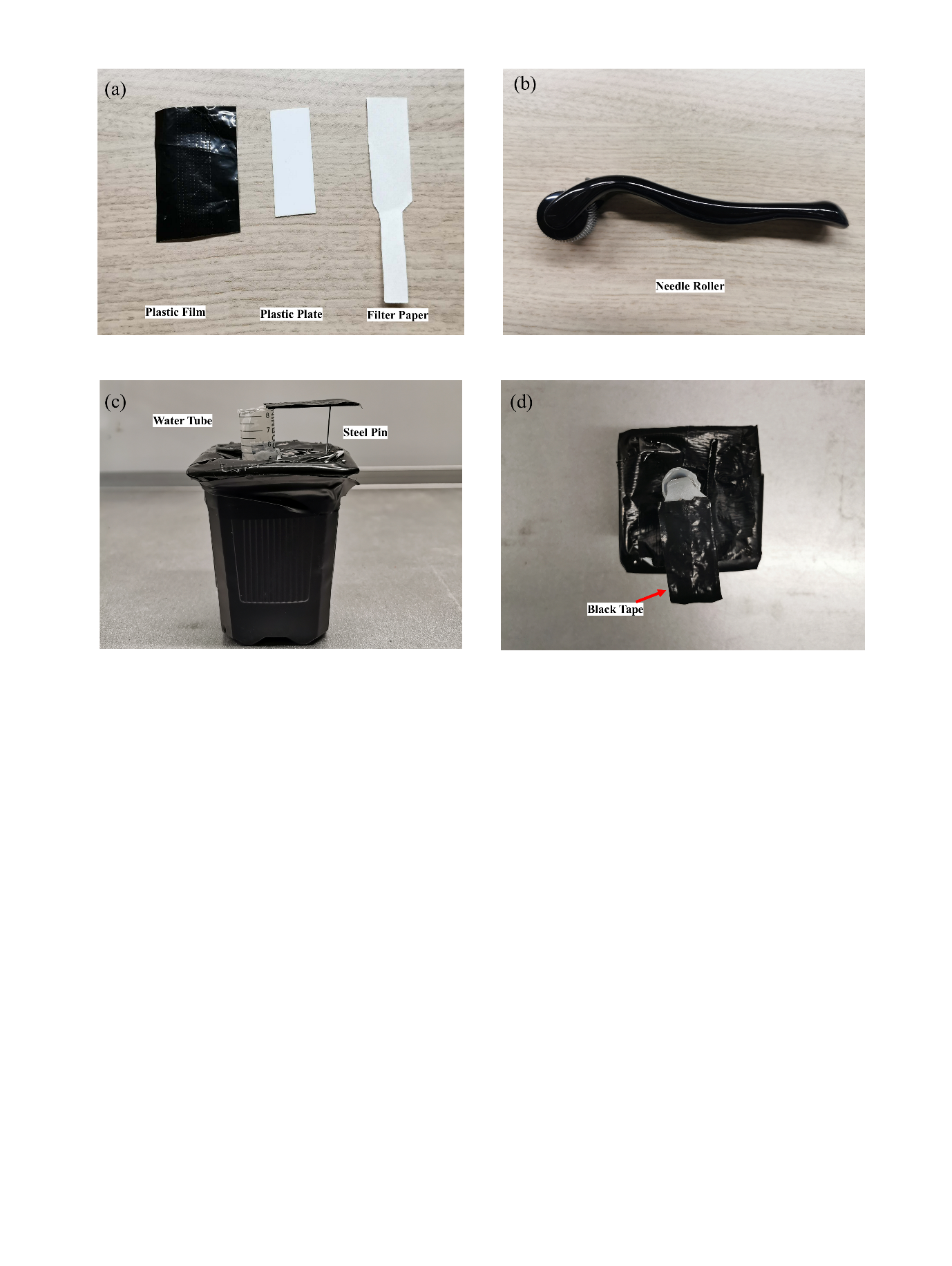
**

**Figure S1.** An overview of the artificial leaves (AL), including the materials, tools, and perspectives from various angles. (a) Components of AL, (b) needle roller with 1 mm needle length used to create pores in the plastic film, (c) side view, and (d) top view of AL in the experimental set-up. The ‘tail’ of the AL was placed in a tube filled with water and supported by a steel pin.

**
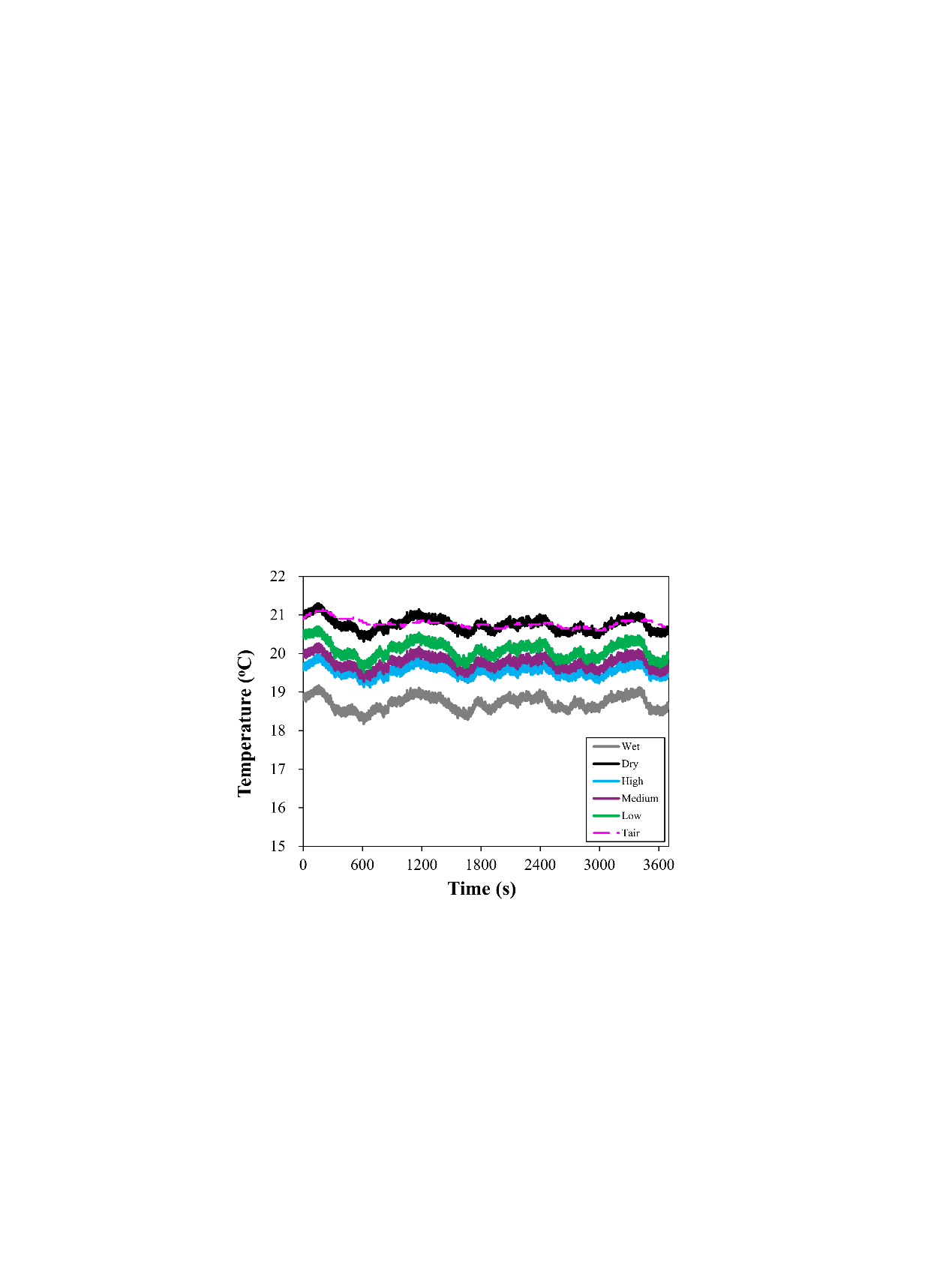
**

**Figure S2.** Temperature kinetics of five artificial leaves (wet, dry, high, medium, and low), as well as air temperature (T_air_), when left in the dark for >1 h.





**Figure S3.** Time courses of pore conductance to water vapour (g_pw_) of three artificial leaves (AL) in the dark, as determined by gas exchange (Li-6800). (a) Low g_pw_, (b) Medium g_pw_, and (c) High g_pw_. Each line represents a different replicate.


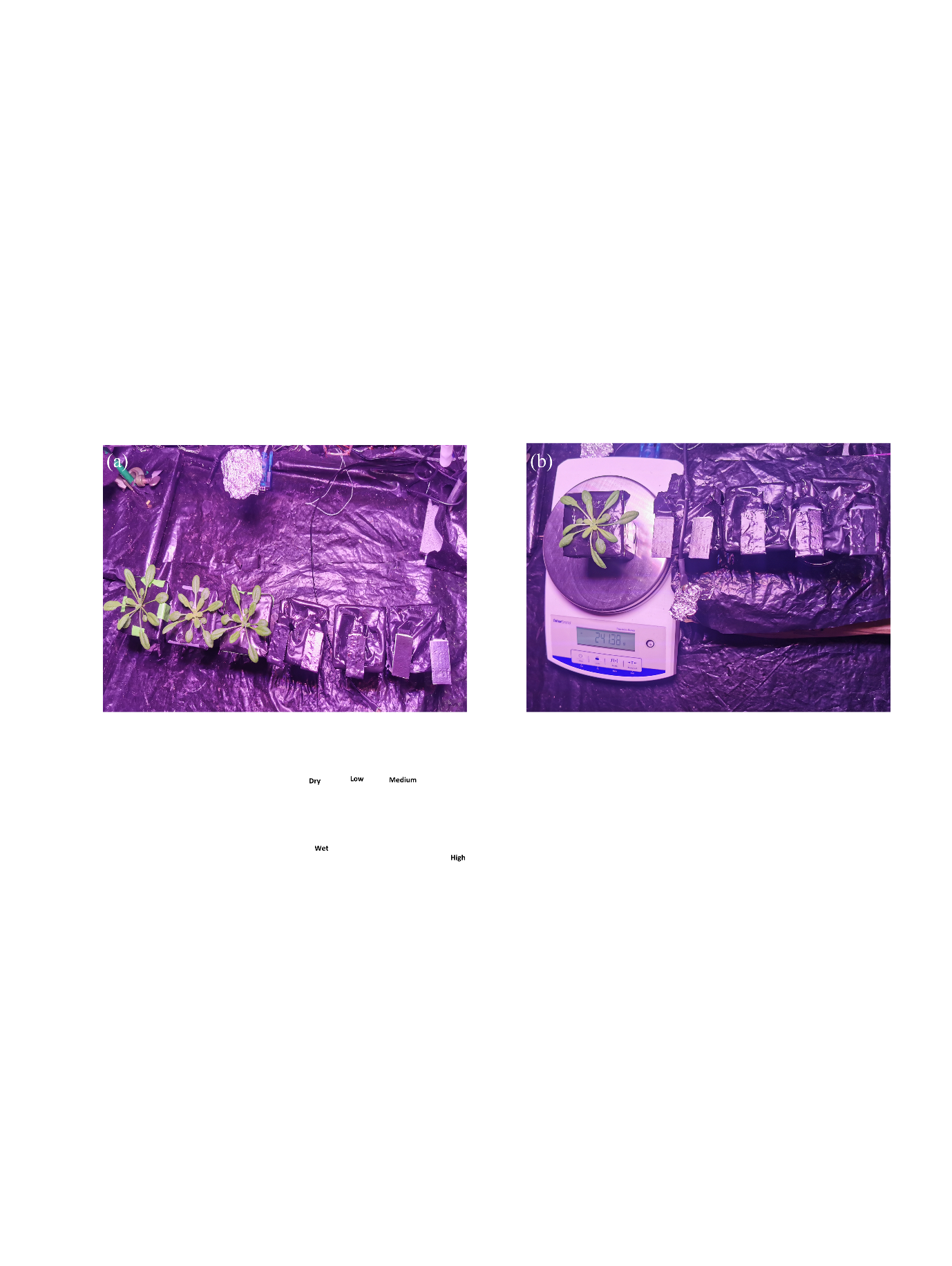


**Figure S4.** Overview of experimental setup for distinguishing different genotypes, and DynG validation by the lysimetric method. (a) three Arabidopsis genotypes (From left to right: *epf1epf2*, Col-0, and EPF2OE) were placed close to five AL (From left to right: low, medium, high, wet, and dry). A crumpled sheet of aluminium foil near the target object was set to ε=1, to quantify the longwave radiation received from surrounding objects, (b) Arabidopsis Col-0 placed on a balance to track weight changes, with five AL near the balance to apply DynG.

**
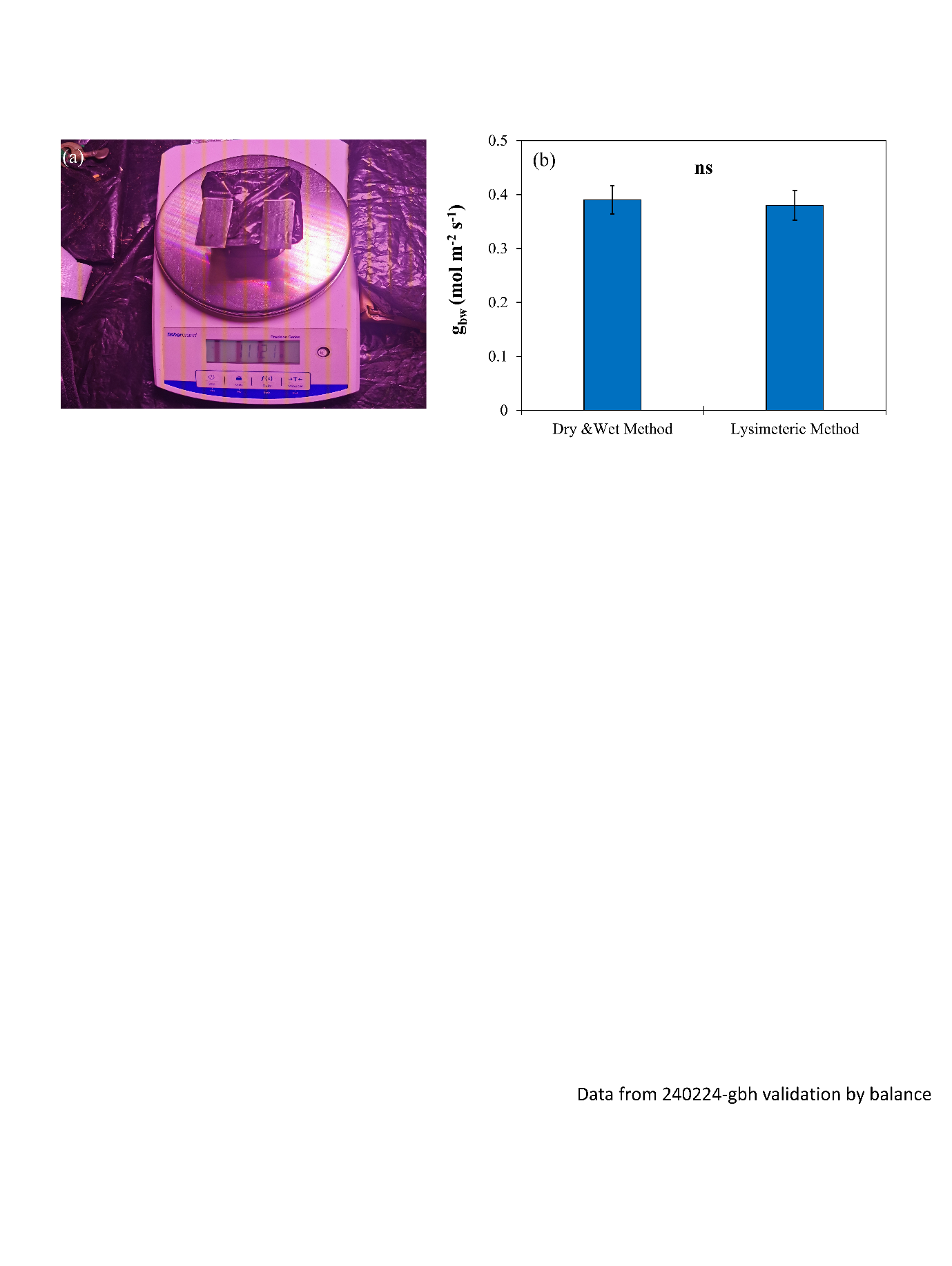
**

**Figure S5.** Validation of method to determine boundary layer conductance to water vapour (g_bw_) in AL. (a) Picture of experimental setup: dry and wet AL were placed in the same pot, which was positioned on a balance to track weight changes caused by evaporation from wet AL. (b) Comparison of g_bw_ by using the ‘Dry & Wet’ method (Eq. 10) and lysimetric method (Eq. 11). Bars show means ± SE (n=3); ns, absence of statistically significant difference (P > 0.05) calculated using a Student’s t-test for paired samples.


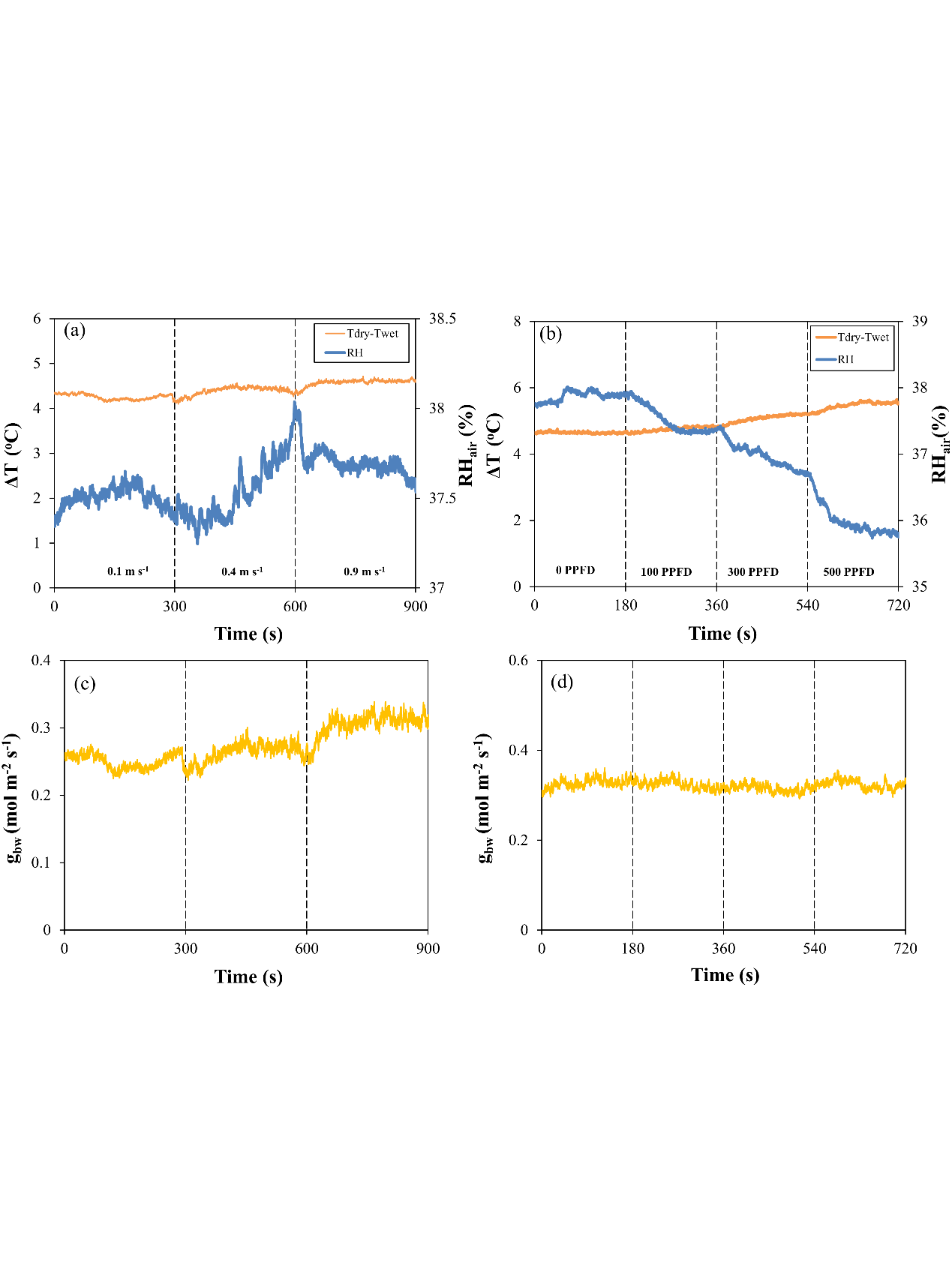


**Figure S6.** Factors underlying DynG under environmental changes. (a, b) Temperature differences between dry and wet AL (T_dry_-T_wet_) as well as air humidity (RH), and (c, d) changes of boundary layer conductance to water vapour (g_bw_). (a) and (c) show effects of wind speed, whereas (b) and (d) show effects of PPFD. Vertical dotted lines show times at which a change in these factors occurred. Values of g_bw_ represent a single ‘leaf’ side and were calculated from Eq. 10 with dry and wet references.





**Figure S7.** Comparison of transpiration rate (E) of Arabidopsis Col-0 by using lysimetric and DynG methods (Eq.7) at low wind speed (~0.2 m s^-1^).

**Dataset S1.** Spreadsheet for calculating g_sw_ based on DynG.
